# Locoregional immunotherapy of malignant effusion from colorectal cancer using the streptococcal preparation OK-432 plus Interleukin-2: Induction of autologous tumour-reactive CD4+ Th1 killer lymphocytes

**DOI:** 10.1038/sj.bjc.6601379

**Published:** 2003-11-11

**Authors:** Y Yamaguchi, E Miyahara, A Ohshita, Y Kawabuchi, K Ohta, K Shimizu, K Minami, J Hihara, A Sawamura, T Toge

**Affiliations:** 1 Department of Surgical Oncology, Research Institute for Radiation Biology and Medicine, Hiroshima University, Japan

**Keywords:** OK-432, IL-2, malignant effusion, colorectal cancer, T-cell receptor (TCR), clonotypic PCR

## Abstract

In total, 16 patients with cytologically proven malignant effusion from colorectal cancer were treated by locoregional administration of the streptococcal preparation OK-432 alone or OK-432 plus the T-cell growth factor interleukin (IL)-2, and the action mechanism of the treatment was studied. A positive clinical response, showing a cytologic disappearance of cancer cells and decrease of effusion, was observed in nine of 11 (82%) patients treated with OK-432 alone and in all five patients treated with OK-432 plus IL-2. Flow cytometric analysis revealed that OK-432 plus IL-2 locally induced acute inflammation-like responses, including serial cellular infiltrations of granulocyte migration within a matter of hours, and activation of macrophages and T lymphocyte involvement within the following days, and that a predominant expansion of CD3+CD4+ lymphocytes (CD: cluster of differentiation) was induced by *in vitro* stimulation with IL-2 of locoregional cells after the OK-432 administration (OK/IL-2AK cells). The OK/IL-2AK cells produced tumour necrosis factor-*α* and interferon-*γ*, but these cells did not produce IL-4 and IL-6. The OK/IL-2AK cells expressed potent killing activity against autologous tumour cells. This activity was abrogated by treatment of the lymphocytes with anti-CD3, -CD4, -TCR*αβ* antibody, and by the treatment of target cells with anti-human leukocyte antigen (HLA)-DR antibody. The OK/IL-2AK cells expressed Fas-L gene, and flow cytometric analysis demonstrated HLA-DR expression in approximately 75% of CEA+ or cytokeratin+ effusion cells. TCRV*β* gene analysis of the OK/IL-2AK cells showed an oligoclonal usage of TCR*β*20, which was also involved in the cytotoxic mechanism of the OK/IL-2AK cells. Single-strand conformational polymorphism analysis demonstrated the clonotypes for the TCRV*β*20 gene, and the CDR3s of the gene were sequenced. The clonotypic PCR using the TCRV*β*20-CDR3 sequences could detect the CDR3-identical TCRs in effusion lymphocytes from the other patients. Taken together, it is suggested that locoregional administration of OK-432 plus IL-2 is highly effective for the management of malignant effusion from colorectal cancer. OK-432 plus IL-2 induces autologous tumour-reactive CD4+ Th1 killer lymphocytes, which recognise tumour antigen(s) presented with HLA class II molecules on effusion tumour cells by means of preferential usage of TCRV*β*20. The clonotypic PCR using the TCRV*β*20-CDR3 sequences may be informative for treating malignant effusion from colorectal cancer using OK-432 plus IL-2.

Malignant effusion is often a complication in primary or refractory cancer patients. Malignant effusion is a poor prognostic factor; 20% of patients die within 1 month, and the median survival is only a few months (Miles and Knight, 1993). When the effusion is diagnosed, 75% of patients have several objective and subjective symptoms such as anorexia, a full abdominal sensation, cough, and dyspnoea, which are sometimes difficult to be managed. Locoregional administration of various agents, including antineoplastic chemotherapeutic drugs (Kimura *et al*, 1998), biological response modifiers for immunotherapy (Driesen *et al*, 1994), and gene therapy agents (Hsiao *et al*, 1997) by paracentesis may be effective for the treatment of malignant effusion due to the pharmacokinetic advantages of drug delivery.

OK-432 is one of the most popular anticancer drugs and is the only agent approved for locoregional use in patients with malignant effusion in Japan (Nio *et al*, 1999; Gochi *et al*, 2001). It is a penicillin- and heat-inactivated lyophilised powder of *Streptococcus pyogenes* A3. OK-432 modifies host cellular immune responses biologically to potentiate antitumour activities. The mechanisms responsible for the antitumour activity of OK-432 have previously been characterised. Uchida and Micksche (1983) demonstrated that intrapleural administration of OK-432 augmented the effusion of natural killer (NK) cell activity and reduced the effusion of NK suppressor cell activity. Katano and Torisu (1983) reported the induction of tumoricidal neutrophils by intracavitary administration of OK-432. Moreover, OK-432 has been reported to activate tumoricidal macrophages (Uchida and Klein, 1985) and to involve T cells (Ozaki *et al*, 1990).

We have investigated locoregional immunotherapy for malignant effusion, and have previously reported the clinical efficacy of the combined administration of OK-432 plus the T-cell growth factor interleukin-2 (IL-2) in gastric cancer patients. In that report, we suggested that the efficacy of locoregional immunotherapy for malignant effusion using OK-432 plus IL-2 was dependent on an induction of CD4+ effector lymphocytes (CD: cluster of differentiation) (Yamaguchi *et al*, 1995). In the present study, we demonstrated a high efficacy of the locoregional immunotherapy using OK-432 alone or OK-432 plus IL-2 for malignant effusion from colorectal cancer. Furthermore, locoregional cells were analysed in order to clarify the mechanisms involved in the locoregional immunotherapy using OK-432 plus IL-2.

## MATERIALS AND METHODS

### Locoregional immunotherapy for malignant effusion using OK-432 plus IL-2

Since 1996, 16 colorectal cancer patients with cytologically proven malignant effusion were experienced and treated with OK-432 alone or with OK-432 plus IL-2 in our department, after patients gave informed consent (Yamaguchi *et al*, 1995). Briefly, 0.2–5 KE of OK-432, which was dissolved in 10 ml of saline, was locoregionally administered at the time of paracentesis after the removal of the effusion, and was followed by administration of 100 000 IU of IL-2 (TGP-3, Takeda Pharmaceutical Co., Ltd., Osaka) in saline 3–7 days later. The clinical efficacy of the treatment was assessed by cytological, X-ray and computed tomographic examinations before and 1 month after the treatment, and a positive response was defined when effusion had either disappeared or decreased, with negative cytology results persisting for more than 1 month. Sonographic examinations were made weekly to address the assessment of clinical responses. All patients had already received concurrent chemotherapy of 5-fluorouracil (600 mg m^−2^, weekly) plus leucovorin (250 mg m^−2^, weekly), which had failed to inhibit the effusion.

### Collection of effusion lymphocytes and tumour cells

Citrated effusion was obtained before OK-432 administration. A portion of the effusion was subjected to a whole effusion assay, described below. Another portion of the effusion was processed in order to collect effusion cells. Cells were pelleted and resuspended in RPMI-1640 medium and layered on a 75%/100% Ficoll–Conray gradient. After centrifugation at 800 **g** for 25 min, autologous tumour cells were collected from the 75% interface and mononuclear cells were collected from the 100% interface. Tumour cells were washed three times with RPMI-1640 medium and were frozen in fetal bovine serum supplemented with 10% DMSO until they were used as a target for the cytotoxicity assays. After OK-432 administration, the effusion mononuclear cells were collected again, washed, and further incubated in RPMI-1640 medium supplemented with 2% heat-inactivated autologous serum and 100 U ml^−1^ IL-2 for 7–21 days. The OK-432 and IL-2-activated killer (OK/IL-2AK) cells were harvested and subjected to further experiments. Human leukocyte antigen (HLA) haplotypes of the patients were serologically measured at SRL Inc., Tokyo.

### Flow cytometry (FCM) and monoclonal antibodies

Effusion cells (50 *μ*l) before and after the treatment and OK/IL-2AK cells (5 × 10^5^) were incubated with fluorescein isothiocyanate (FITC) or phycoerythrin (PE)-labelled monoclonal antibodies at 4°C for 45 min. The monoclonal antibodies used were anti-Leu4a (CD3), -Leu3a (CD4), -Leu2a (CD8), -Leu19 (CD56), -Leu15 (CD11b), -HLA-DR, - T-cell receptor (TCR)*αβ* (Becton Dickinson, San Jose, CA, USA), -TCRV*β*20, -HLA-ABC, (Immunotech, Marseille, France), -carcinoembryonic antigen (CEA) (DAKO, A/S, Denmark), -cytokeratin AE1/AE3 (DAKO Co., CA, USA) antibodies. To determine the HLA-DR expression in tumour cells, effusion cells (5 × 10^5^) were incubated with anti-CEA or -cytokeratin antibody followed by PE-labelled rat anti-mouse Ig*κ* light-chain antibody (BD Biosciences, Tokyo); cells were then washed three times and stained with FITC-labelled anti-HLA-DR antibody. Finally, the cells were washed twice and resuspended in phosphate-buffered saline. Flow cytometric analysis was performed using a Cytoron (Ortho Diagnostic Systems, USA). After the cells were adequately gated by using a forward and side scatter, data collection was set up to stop when 10 000 events had been analysed.

### Cytotoxicity assay

The cytotoxic activity of the lymphocytes was determined using a standard ^51^Cr releasing assay. In brief, ^51^Cr-labelled autologous tumour cells (5 × 10^3^) and various numbers of effector lymphocytes were cocultured in 96-well round-bottomed microtitre plates at a volume of 200 *μ*l. After 4 h of incubation at 37°C, the radioactivity of the supernatants was measured using an autogamma scintillation counter (500C, Packard, USA). Spontaneous release was determined in wells containing the target cells alone, and maximum release was obtained by adding 100 *μ*l of 1% Triton X-100 solution instead of the effector cells over the target cells. Cytotoxic activity was calculated from triplicate samples using the following formula: cytotoxic activity (%)=(experimental release (cpm)−spontaneous release (cpm))/(maximal release (cpm)−spontaneous release (cpm)) × 100. To explore the lymphocyte phenotype and molecules involved in the cytotoxic mechanism, either effector cells or target cells were treated with the corresponding antibodies at 4°C for 1 h, washed, and then subjected to cytotoxicity assay.

### Cytokine assay

A simple whole effusion assay, which imitated *in vivo* treatment, was performed to predict the responsiveness of effusion to OK-432 treatment before the onset of treatment. In brief, aliquots of effusion (0.5 ml) were directly diluted with RPMI-1640 medium (4.5 ml) and stimulated with 0.1 KE ml^−1^ of OK-432. After 24 h under standard culture conditions, the supernatants were collected and tumour necrosis factor (TNF)-*α* production was measured using an ELISA kit (R & D Systems, Minneapolis, MN, USA).

A profile of cytokine production by OK/IL-2AK cells was also measured by enzyme-linked immunosorbent assay (ELISA). Aliquots of OK/IL-2AK cells were collected, washed, and resuspended in RPMI-1640 medium containing IL-2 at a cell density of 10^6^ ml^−1^. After being incubated for another 48 h, the culture supernatants were collected by centrifugation, and the production of interferon (IFN)-*γ*, TNF-*α*, IL-4, and IL-6 was measured using an ELISA kit specific for each cytokine (R & D Systems, Minneapolis, MN, USA).

### Reverse transcription–polymerase chain reaction (RT–PCR) analysis for Fas-L expression

RT–PCR was performed in order to analyse the Fas-L gene. In brief, total cellular RNA was extracted by acid guanidinium thiocyanate–phenol–chloroform extraction, and RNA samples were reverse-transcribed into cDNA with a random hexamer (Miyahara *et al*, 1999). The PCR amplification of the cDNA was performed in a reaction mixture consisting of cDNA samples, *Taq* polymerase (Gibco BRL, USA) and the following primers: Fas-L, 5′-ATAGGATCCATGTTTCTGCTCTTCCACCTAC AGAAGGA-3′, and 5′-ATAGAATTCTGACCAAGAGAGAGCTCAGATA CGTTGAC-3′ (Yasukawa *et al*, 1999); *β*-actin (Stratagene, La Jolla, CA, USA). The reaction was carried out on a Perkin-Elmer Cetus thermal cycler, under conditions involving a 3-min denaturation at 94°C followed by 35 cycles of 1 min at 94°C, 1 min at 55°C, and 1 min at 72°C. After amplification, 8 *μ*l of the reaction mixture was removed and analysed by means of electrophoresis through 2.0% agarose gels in Tris-borate-EDTA buffer, and the gels were then stained with ethidium bromide. The expected lengths of the amplified cDNAs were 506 and 514 bp for Fas-L and *β*-actin, respectively.

### TCRV*β* gene analysis by RT–PCR–Southern blotting

TCRV*β* gene analysis by RT–PCR was performed as above mentioned using primers specific for TCRV*β* genes, which have been previously described in detail (Miyahara *et al*, 1996). The amplified DNA was confirmed by Southern blot analysis using the C*β* sequence with luminous reaction. Briefly, 8 *μ*l of each amplified product was run on a 1% agarose gel and transferred onto a nylon membrane (Hybond-N+, Amersham International plc, Aylesburg, UK). The membranes were hybridised at 42°C for 4 h with 10 ng ml^−1^ of probe specific for C*β* forward, which had been labelled at its 3′ end with fluorescein-d UTP (ECL 3′-oligolabelling and detection systems, Amersham International plc, Aylesburg, UK). After incubation with the antifluorescein HRP, hybridised probes were detected by the light (*λ*_max_, 428 nm) emitted from the oxidation of luminol-coupled peroxidase. The light output was detected on X-ray film.

### Diagnostic SSCP analysis

To detect single-base mutations in the PCR products of the TCRV*β* gene, the single-strand conformation polymorphism (SSCP) technique was used (Orita *et al*, 1989). In brief, 5 *μ*l of the asymmetric PCR product, which was mixed with 5 *μ*l of 95% formamide containing xylen cyanol and bromophenol blue, was heated at 95°C for 5 min, cooled on ice, and loaded onto a 10% acrylamide mini-gel (Mino-Protean II, Bio-Rad, Hercules, CA, USA). This gel was run at 100 V for 4 h in a cold room (4°C). The gel was then silver stained (Silver Stain Plus, BioRad, Hercules, CA, USA).

### Sequencing of TCR V*β* gene and clonotypic PCR

After SSCP analysis, the amplified DNA was eluted from the acrylamide gel and either directly sequenced in the Gene Analysis Center (Takara Shuzo Co., Ltd, Ohtsu) or cloned in the pCRTM vector, according to the TA cloning kit procedure (Invitrogen, San Diego, CA, USA), and the DNA was subsequently used to transform *Esherichia coli*-competent cells. Plasmid DNA of recombinant colonies was prepared (Wizard, QIAGEN Inc., Chatsworth, CA, USA) and sequenced using the DyeDeoxy Terminator Cycle sequencing kit and 373 DNA Sequencer (Applied Biosystems Inc, Foster City, CA). Definition of complementarity-determining region (CDR) 3-like boundaries was accomplished using methods described by Toyonaga *et al* (1985). CDR3 oligonucleotides were chosen with the help of the Oligo 4.0 computer program and were synthesised by Biologica Inc (Nagoya). Clonotypic PCR was performed using each oligonucleotide as the forward primer and C*β* reverse primer, followed by Southern blot hybridisation with the probe specific for the C*β* forward sequence.

### Statistical analysis

Statistical evaluation of the experimental values was performed using paired and unpaired Student's *t*-test.

## RESULTS

### Characteristics of patients enrolled in locoregional immunotherapy of malignant effusion using OK-432

The characteristics of the 16 patients included in the present study are listed in Table 1

**Table 1:** Characteristics of colorectal cancer patients enrolled in the trial of locoregional immunotherapy for malignant effusion

Age: median (range)	59 (39–72)
Gender (male/female)	7/9
Performance status (0/1/2/3)	1/3/9/3
Primary site (A/T/S/R)^a^	3/2/7/4
Primary operation (yes/no)	14/2
*Histology*	
Differentiated type^b^	14
Undifferentiated type^c^	2
	
*Effusion*	
Pleural	3
Ascites	13
	
*Concurrent metastasis*	
Liver	10
Lung	3
Lymph nodes	12
Bone	2

a A=ascending; T=transverse; S=sigmoid; R=rectum.

b Differentiated type included well to moderately differentiated adenocarcinomas.

c Mucinous adenocarcinoma.

. The patients had a median age of 59 ranging from 39 to 72 years old, included seven males and nine females, and showed PS values of 0–3. Primary lesions were located at ascending, transverse, sigmoid colons, and rectums. A total of 14 patients had undergone prior resection of the primary tumour. In total, 14 patients had well to moderately differentiated adenocarcinomas and two patients did mocinous ones in histology of the primary lesions. In all, 13 patients had ascites and three did pleural effusion, and all patients had concurrent liver, lung, lymph node, and bone metastases as well as effusions detectable by CT scan.

### Clinical response of locoregional treatment for malignant effusion using OK-432

The clinical efficacy of locoregional immunotherapy using OK-432 or OK-432 plus IL-2 is shown in Table 2

**Table 2:** Clinical responses of locoregional immunotherapy using OK-432 alone or OK-432 plus IL-2 for malignant effusion from colorectal cancer

	**Immunotherapy**		
**Efficacy**	**OK-432**	**OK-432+IL-2**	**Total**
*Response (%)*	9/11 (82)	5/5 (100)	14/16 (88)
Disappeared	7 (64)	4 (80)	11 (69)
Decreased	2 (18)	1 (20)	3 (19)
			
*Duration (months)*			
Median	6	8	8
Range	1–16	3–16	1–16
			
*Suvival (months)*			
Median	6	8	8
Range	1–21	3–19	1–21

. Initially, 11 patients were treated with OK-432 alone and another five patients were done with OK-432 plus IL-2. The response rate of locoregional immunotherapy for malignant effusion was 82% with OK-432 alone and 100% with OK-432 plus IL-2, showing the overall response rate of 88%. Subsequently, symptoms due to malignant effusion appeared to improve in responder patients (data not shown). No patients except two had a clinical relapse of effusion after the immunotherapy using OK-432 or OK-432 plus IL-2. The survival of each treatment group was similar, and the overall median survival was 8 months ranging from 1 to 21 months. As adverse effects, all patients had fever elevation of 37–38°C and four patients (25%) had abdominal or thoracic pain, both of which could be well-managed with an occasional administration of nonsteroidal anti-inflammatory drugs (data not shown).

### Cytograms for locoregional cells infiltrated with the OK-432 plus IL-2 treatment

**Figure 1:**
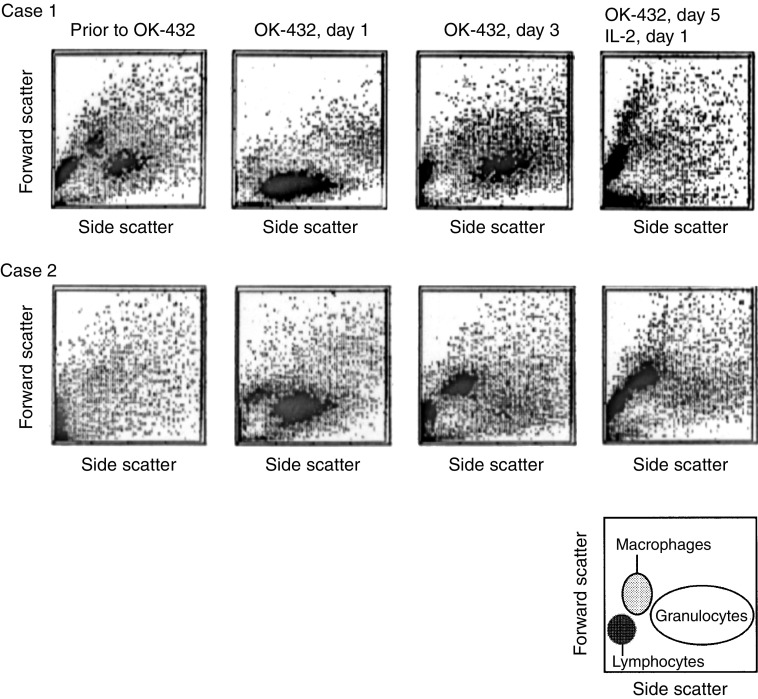
Serial cellular infiltration after administration of OK-432 plus IL-2. OK-432 and IL-2 were administered into malignant effusion and locoregional cells were analysed by FCM on the days indicated. Representative cytograms obtained from two patients with malignant effusion were shown.

Effusion cells were collected before and after OK-432 plus IL-2 administration, and cytogram analysis was performed using FCM to determine the changes in the infiltrated cell population. Representative cytograms obtained from two patients with malignant effusion are shown in Figure 1

. Granulocytes, the population of which was found to have high side scatter and intermediate forward scatter, migrated into the cavity in great numbers even 4 h after the administration of OK-432. This population peaked on day 1, and then decreased. This granulocyte mobilisation was closely correlated clinically with the febrile condition of the patients after the OK-432 administration. Macrophages, which had intermediate side scatter and large forward scatter, followed the granulocyte accumulation into the cavity. Lymphocytes, which had low side scatter and small forward scatter, accumulated gradually after the OK-432 administration and were remarkably concentrated by IL-2 administration *in vivo*.

### Phenotype analysis and cytokine production of OK/IL-2AK cells

**Figure 2:**
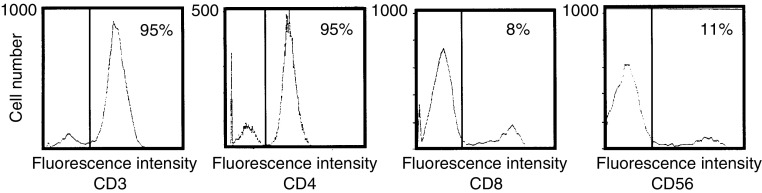
Phenotype analysis of OK/IL-2AK cells. Effusion lymphocytes were collected after OK-432 administration and stimulated *in vitro* with IL-2 for 7–21 days. Cells were subjected to phenotype analysis using Cytoron.

Effusion cells after OK-432 administration were collected and further stimulated *in vitro* with IL-2 (OK/IL-2AK cells) and characterised by FCM. The representative profile is shown in Figure 2

. The OK/IL-2AK cells were 95% CD3+CD4+, but were only 8% CD8+ and 11% CD56+ on day 14 of the culture. Similar phenotypic changes were obtained in the OK/IL-2AK cells from the three patients who were examined. Culture supernatants of the OK/IL-2AK cells were also collected, and cytokine production was examined using ELISA (Table 3

**Table 3:** Cytokine production profiles of OK/IL-2AK cells

**Cytokines**	**Case 1**	**Case 2**	**Case 3**
IFN-*γ* (U ml^−1^)	2000	1044	954
TNF-*α* (pg ml^−1^)	3810	2710	883
IL-4 (pg ml^−1^)	8	12	<4
IL-6 (pg ml^−1^)	3	20	11

Effusion cells were collected after OK-432 administration and stimulated *in vitro* with IL-2. OK/IL-2AK cells (10^6^ ml^−1^) were cultured for 48 h and cytokine production in supernatant was evaluated using ELISA.

). The OK/IL-2AK cells (10^6^ ml^−1^) produced high levels of IFN-*γ* (954–2000 U ml^−1^) and TNF-*α* (883–3810 pg ml^−1^), but only marginal levels of IL-4 and IL-6 were produced during 48 h in the presence of IL-2. Effusion cells before OK-432 administration showed a diverse pattern of cytokine production; IFN-*γ* and TNF-*α* were marginally detectable, and IL-6, on the other hand, was sometimes high at a concentration (data not shown).

### Cytotoxic property of OK/IL-2AK cells

A cytotoxicity assay was performed to explore the cytotoxic properties of this predominant CD4+ population (Table 4

**Table 4:** Cytotoxic property of OK/IL-2AK cells

**Treatment**		**Autologous tumour cell target**		**K562 cell target**	
**Effector cells**	**Target cells**	**Pre-OK**	**OK/IL-2AK**	**Pre-OK**	**OK/IL-2AK**
—	—	42±12	84±3	52±4	62±1
Control Ig	—	47±8	83±12	51±5	60±2
Anti-CD3	—	—	32±15**	—	—
Anti-CD4	—	—	37±11**	—	—
Anti-CD8	—	—	89±7	—	—
Anti-CD56	—	—	72±7*	—	—
Anti-TCR *αβ*	—	21±6*	41±4**	48±4	59±2
Anti-TCRV *β* 20	—	40±5	49±2**	51±3	62±3
					
—	Control Ig	—	79±12	—	—
—	Anti-HLA-ABC	—	74±8	—	—
—	Anti-HLA-DR	—	36±7***	—	—

Effusion cells were collected and cultured as described in Materials and Methods. Cytotoxic activity against autologous tumour cells and K562 tumour cells was investigated with the treatment of effector cells and target cells with antibodies indicated. *E*/*T*=100. Significant differences from the value without treatment,

* *P*<0.05,

** *P*<0.01.

). The OK/IL-2AK cells, obtained from a rectal cancer patient with an excellent effusion response, showed potent cytotoxic activity (84%) against autologous tumour cells at an effector-to-target (E/T) ratio of 100. This activity appeared to be higher than that observed before the treatment (pre-OK). The cytotoxicity of the OK/IL-2AK cells was almost completely abrogated by the treatment of effector cells with either anti-CD3 (32%) or -CD4 (37%) antibody prior to the cytotoxicity assay. The cytotoxicity was not affected at all by anti-CD8 antibody, and was only slightly affected by anti-CD56 antibody (72%). The cytotoxicity was also reduced when the OK/IL-2AK cells were treated with anti-TCR*αβ* antibody (41%), and when tumour cells were treated with anti-HLA-DR antibody (36%). However, treatment of the effector cells with irrelevant Ig had no effect on the cytotoxicity generated. When an irrelevant target cell line, K562, was used in the cytotoxicity assay, no remarkable augmentation of the cytotoxicity of the OK/IL-2AK cells was observed, nor was there an inhibitory effect with the use of anti-TCR*αβ* antibody.

### Fas-L expression in OK/IL-2AK cells

**Figure 3:**
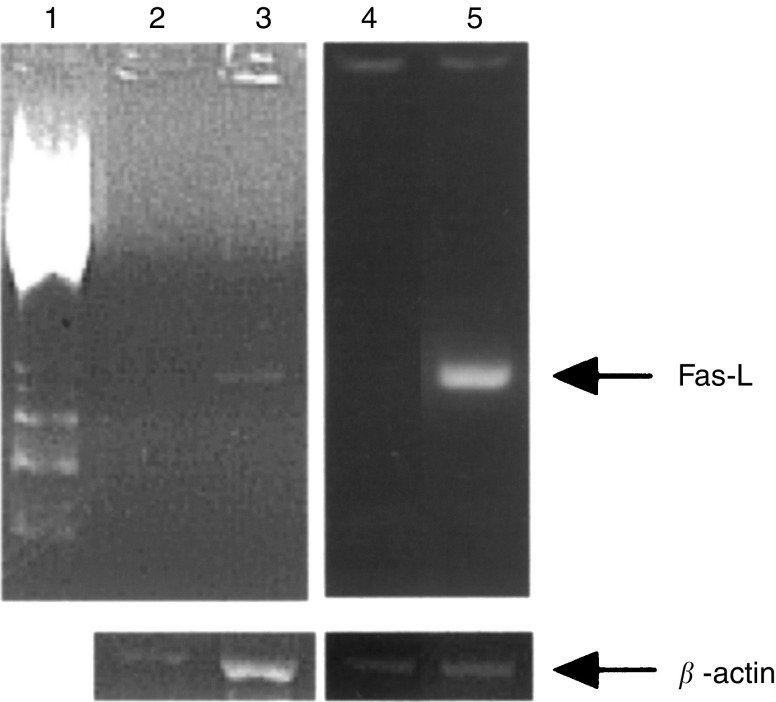
Fas-L gene expression of OK/IL-2AK cells. mRNA was extracted from effusion cells before OK-432 administration and OK/IL-2AK cells, and Fas-L gene expression was investigated by RT–PCR analysis using specific primers. Lane 1, molecular marker; lane 2 and 4, cells before OK-432 administration; lanes 3 and 5, OK/IL2AK cells.

Fas-L expression was examined using RT–PCR for the OK/IL-2AK cells. Representative data are shown in Figure 3

. We were unable to detect positive bands of Fas-L expression in the effusion cells before OK-432 administration. However, the OK/IL-2AK cells showed positive bands for Fas-L expression in patients analysed.

### HLA-DR expression in effusion tumour cells

The HLA-DR expression in effusion tumour cells was examined by FCM (Table 5

**Table 5:** HLA-DR expression on CEA+ or cytokeratin+ effusion cells

**Surface antigens**	**Case 1**	**Case 2**	**Case 3**	**Case 4**
CEA+	7^a^	38	45	75
Cytokeratin+	19	34	50	66
CD3+	57	20	41	3
HLA-DR+	8	87	47	82
				
HLA-DR+CEA+	5 (72^b^)	27 (71)	37 (81)	68 (91)
HLA-DR+cytokeratin+	6 (30)	34 (100)	39 (78)	63 (95)

Effusion cells were collected before the treatment and stained with antibodies indicated. Flow cytometric analysis was performed on Cytoron.

a Percentage of surface antigen-positive cells in effusion cells analysed.

b HLA-DR+ cells in CEA+ or cytokeratin+ cells.

). HLA-DR expression was detectable in 8–87% of the effusion cells, as determined by single colour analysis. CEA+ cells and cytokeratin+ cells were detected in 7–75 and 19–66% of the effusion cells, respectively. Two-colour analysis showed that 5–68% of the effusion cells were of the HLA-DR+CEA+ phenotype and 6–63% of the cells were of the HLA-DR+cytokeratin+ phenotype, indicating that approximately 75% of the CEA+ cells or cytokeratin+ cells expressed HLA-DR molecules on their cell surface.

### TCRV*β* gene usage analysis of OK/IL-2AK cells

**Figure 4:**
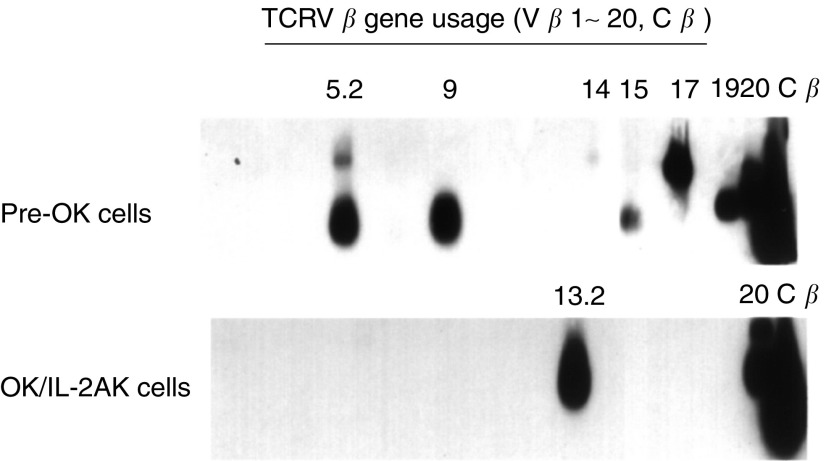
TCRV*β* gene analysis on OK/IL-2AK cells. Effusion cells were collected before and after OK-432 administration from a rectal cancer patient with malignant effusion (HLA-A11,24, B62,51, Cw4, DR9) and TCRV*β* usage was analysed by RT–PCR using primer pairs specific for TCRV*β*1 to 20 and C*β*, followed by Southern blot hybridisation using C*β*-specific probe.

We next analysed the TCRV*β* gene usage of the OK/IL-2AK cells from the rectal cancer patient (Figure 4

). The TCRV*β* gene analysis demonstrated a diverse expression of TCR genes in the effusion cells before OK-432 administration (pre-OK cells). In contrast, an oligoclonal expression of TCRV*β*13.2 and TCRV*β*20 genes was observed in the OK/IL-2AK cells of this patient. Flow cytometric analysis showed that TCRV*β*20+ lymphocytes accounted for 15% of the pre-OK cells and 26% of the OK/IL-2AK cells (data not shown). The cytotoxicity of the OK/IL-2AK cells was significantly reduced when the OK/IL-2AK cells were treated with anti-TCRV*β*20 antibody (49%) (*P*<0.01) (Table 3).

### CDR3 sequences of the TCRV*β*20 gene

**Figure 5:**
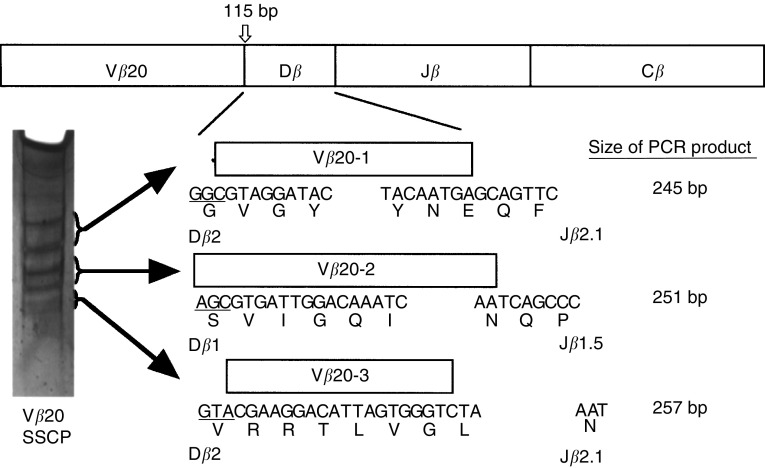
SSCP analysis and CDR3 sequences of TCRV*β*20. PCR products of TCRV*β*20 in Figure 4 were developed on SSCP analysis. Clonotypic bands were sequenced and CDR3 boundaries were determined as indicated.

To identify the CDR3 sequences, the PCR products of TCRV*β* genes from the same patient were examined by SSCP analysis of the TCRV*β*13.2 gene of the OK/IL-2AK cells; this SSCP analysis failed to detect clonotypic bands and demonstrated a smear pattern (data not shown). On the other hand, three pairs of clonotypic bands were detectable in the TCRV*β*20 gene of the OK/IL-2AK cells (Figure 5

). The sequencing analysis demonstrated three CDR3 boundaries, which had diverse regions of D*β*2, D*β*1, and D*β*2 and joining regions of J*β*2.1, J*β*1.5, and J*β*2.1, respectively. There was no similarity among the CDR3 sequences at the nucleotide level, nor at the amino-acid level.

### Clonotypic PCR amplification for detecting CDR3-identical TCRs

**Figure 6:**
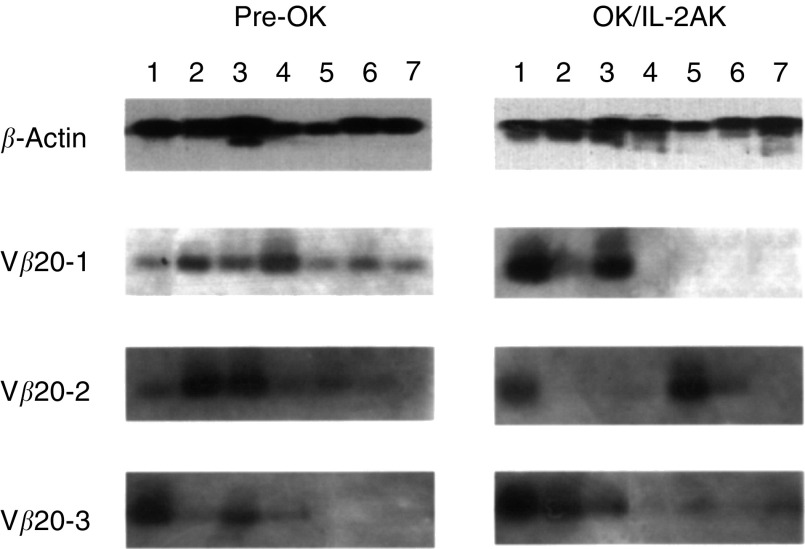
Clonotypic PCR using TCRV*β*20-CDR3 sequences on pre-OK and OK/IL-2AK cells. Clonotypic PCR was performed using CDR3 sequences shown in Figure 5. Lane numbers indicated each patient investigated. HLA haplotypes of patients were as follows: patient 1, HLA-A11,24, B62, 51, Cw4, DR9; patient 2, HLA-A24,33, B62,44, Cw3, DR4,6; patient 3, HLA-A24,31, B54,44, Cw1, DR4,6; patient 4, HLA-A2,10, B35,39, Cw3,7, DR6,8; patient 5, HLA-A11,26, B67,61, Cw7, DR2,9; patient 6, HLA-A2,31, B7,51, Cw7, DR1,12; patient 7, HLA-A24,26, B59,61, Cw1, DR2,4.

According to the sequences of the CDR3 boundaries, we designed forward primers (shown in Figure 5), which were designated as V*β*20-1, -2, and -3, to perform the clonotypic PCR analysis for detecting the TCRs, the CDR3 sequences of which were identical to those cloned from the TCRV*β*20 gene. The expected sizes of the clonotypic PCR products were 245, 251, and 257 bp for V*β*20-1, -2, and -3, respectively (Figure 5). The clonotypic PCR was performed for pre-OK cells and OK/IL-2AK cells in seven patients. Lane 1 indicated the patient from whom the primer sequences of V*β*20-1, -2, and -3 were cloned. Positive bands were detectable at expected sizes in all PCRs of lane 1 from pre-OK cells, and were augmented in those from OK/IL-2AK cells (Figure 6

). Positive bands were also detectable in lanes 2–7 to various extent, indicating that the CDR3-identical TCR-bearing T lymphocytes existed in OK/IL-2AK cells of the other patients.

## DISCUSSION

It was observed that malignant effusion from colorectal cancer decreased or disappeared successfully in more than 80% patients when treated with a simple, easily administered streptococcal preparation OK-432 alone or in combination with T-cell growth factor IL-2. This efficacy seemed better than that in gastric cancer patients, in which the earlier study reported that positive clinical responses were obtained in 60 and 81% when treated with OK-432 alone and with OK-423 plus IL-2, respectively (Yamaguchi *et al*, 1995). This difference may be explained, in part, by the histological types, in which colorectal cancer patients had mainly well to moderately differentiated adenocarcinomas, whereas gastric cancer patients did poorly differentiated adenocarcinomas. Although malignant effusions from colorectal cancer were successfully treated with our immunotherapy, its effects on the distant metastases were marginal and all patients died. Nevertheless, symptoms of patients with malignant effusion was subsequently improved by our simple treatment, indicating that our immunotherapy was important for quality of life of the patients even if they had poor prognoses (Miles and Knight, 1993).

We wanted to understand the mechanism of action of this effective immunotherapy for malignant effusion from colorectal cancer. It was observed using FCM that OK-432 induced locally acute inflammation-like responses, including serial cellular infiltrations of the granulocyte migration in a matter of hours and activation of macrophages with T lymphocyte involvement in the following days. This observation was similar to those included in previous descriptions. Katano and Torisu (1982) reported that all adenocarcinoma cells in malignant ascites disappeared within at least 36 h after OK-432 injection, while the number of intraperitoneal neutrophils increased. These neutrophils had a cytostatic effect on ascites-derived tumour cells *in vitro* (1982). This OK-432-induced neutrophil infiltration and activation may be understood by the property of OK-432 to act as a bacterial preparation.

We focused on the functions and characteristics of the lymphocytes induced with OK-432 plus IL-2 treatment. The property of lymphocytes activated with OK-432 plus IL-2 has not been fully explored, although it has been demonstrated that OK-432-induced lymphokines facilitated the subsequent activation of lymphocytes (Katano *et al*, 1991; Nanjo *et al*, 1991). It was observed that the lymphocytes that infiltrated after OK-432 administration, followed by IL-2 activation *in vitro* (OK/IL-2AK cells), were preferentially CD4+ T cells. This result is comparable to an observed increase in the CD4+ population of responding effusion cells in gastric cancer patients during the treatment (Yamaguchi *et al*, 1995). The OK/IL-2AK cells produced T helper type-1 (Th1) cytokines TNF-*α* and IFN-*γ*, and that Th2 cytokines, that is, IL-4 and IL-6, were not produced under these conditions. Monden *et al* (1992) demonstrated that the intratumoral injection of the mixture of OK-432 and fibrinogen enhanced the regression of colorectal cancer, in which a granulomatous hypersensitive response containing many giant cells was generated in the tumour stroma. That feature highly resembled the delayed-type hypersensitivity reaction, which is associated with Th1 responses. These researchers also established CD4+ T-cell clones, which released a Th1 cytokine TNF-*α*, in colorectal cancer patients treated by immunotherapy using a mixture of OK-432 and fibrinogen (Nagaoka *et al*, 1992). Fujimoto *et al* (1997) reported that OK-432 stimulated the IL-12 production of peripheral blood mononuclear cells and upregulated the Th1 phenotype. Collectively, these findings suggest that OK-432 plus IL-2 stimulation induces preferentially the CD4+ Th1 lymphocytes. We have reported previously that in the murine system, there exists a tumour-derived IL-10-associated Th1 disorder in the microenvironment of malignant effusion, and that this disorder is overcome by the locoregional administration of OK-432 (Hihara *et al*, 1999). The Th1 disorder might occur in cases of malignant effusion from colorectal cancers, and the locoregional upregulation of the Th1 population by OK-432 plus IL-2 may result in positive clinical responses in the treatment of malignant effusions. The upregulation of the local Th1 population by OK-432 plus IL-2, however, failed to elicite systemic antitumour immune responses for the distant metastases, since the systemic Th1 disorder might exist in such terminally ill patients with malignant effusion from colorectal cancer (Shibata *et al*, 2002).

Notably, it was observed that the CD4+ OK/IL-2AK cells expressed cytotoxic activity against autologous tumour cells and acquired Fas-L expression. Ozaki *et al* (1990) first described OK-432-induced L3T4+ tumoricidal T lymphocytes in a murine system. It has been established that certain CD4+ T cells can express cytotoxic activity, which is associated with the Th1 phenotype and mediated with the Fas–Fas-L interaction (Hahn *et al*, 1995). Stalder *et al* (1994) have also reported that Fas-L is an effector molecule in CD4+ T-cell-mediated cytotoxicity, in which the transfection of Fas antigen into target cells renders the transfectants more susceptible to the CD4+ CTLs. Taken together, it is strongly suggested that OK-432 plus IL-2 stimulation induces autologous tumour-reactive CD4+ Th1 killer lymphocytes. The CD4+ Th1 killer lymphocytes, which may recognise tumour antigens differently from CD8+ cytotoxic T lymphocytes or NK cells, may be a promising candidate for the effector cells of cancer immunotherapy that targets the heterogenous clinical tumours.

It is of interest whether or not TCR and HLA molecules are involved in the cytotoxic mechanism of the autologous tumour-reactive CD4+ Th1 killer lymphocytes induced with OK-432 plus IL-2. It was found that the autologous tumour-killing activity of the OK/IL-2AK cells was significantly reduced by the treatment of the lymphocytes with anti-TCR*αβ* antibody or by the treatment of the autologous tumour cells with anti-HLA-DR antibody; both results indicated that TCR and HLA class II molecules, but not class I molecules, were involved in the cytotoxic mechanism of the OK/IL-2AK cells. Somasundaram *et al* (2000) reported a CD4+ cytotoxic T-lymphocyte clone against malignant melanoma cells, the cytotoxicity of which was restricted with HLA class I molecules. However, Kierstead *et al* (2001) demonstrated that the CD4+ killer lymphocytes recognised antigens presented by HLA class II molecules. Ozaki *et al* (1990) showed that OK-432 was presented by Ia-positive antigen-presenting cells for OK-432-specific anti-tumour effector T cells, which was in agreement with our observations. In addition, flow cytometric analysis of effusion cells using anti-CEA, -cytokeratin, and -HLA-DR antibodies demonstrated the expression of HLA-DR molecules on CEA+ cells and cytokeratin+ cells in the effusion, which indicated the presence of floating cancer cells. The expression of HLA class II molecules has been reported in many tumour cell types, including melanoma (Ericsson *et al*, 2001), lung cancer (Yano *et al*, 2000), and colon cancer (Bustin *et al*, 2001). It has also been reported that HLA class II expression on tumour cells can be upregulated by IFN-*γ* (Sgagias *et al*, 1996; Yano *et al*, 2000). The microenvironment of effusion may induce the expression of HLA-DR molecules on effusion tumour cells. Together, it is suggested that the OK/IL-2AK cells recognise the antigen(s) which is(are) presented in the context of HLA class II molecules on a tumour cell target.

Involvement of TCR in the cytotoxic mechanism of the OK/IL-2AK cells suggests the existence of limited TCR usage in the cells. Finally, we investigated whether a certain TCR usage was involved in OK-432-induced T-cell responses. The RT–PCR–Southern blot analysis of the effusion lymphocytes showed a highly diverse usage of the TCRV*β* gene in this experiment. This finding is, in general, consistent with previous observations (Cole *et al*, 1997). However, in a comparison of results before and after OK-432 treatment, TCRV*β*20 usage was found to be preferentially expressed in the OK/IL-2AK cells (Miyahara *et al*, 1996). OK-432 stimulation failed to expand TCRV*β*20 usage in lymphocytes from healthy volunteers (Miyahara *et al*, 1996). The OK/IL-2AK cells from the patient we analysed in detail revealed an oligoclonal usage of TCRV*β*20. Importantly, the autologous tumour-killing activity of the OK/IL-2AK cells was significantly reduced by the treatment of the lymphocytes with anti-TCRV*β*20 antibody as well as anti-TCR*αβ* antibody. In addition, SSCP analysis of the TCRV*β*20 gene of the OK/IL-2AK cells showed clonotypic bands. These findings strongly indicate that the TCRV*β*20 was responsible for the cytotoxicity of the OK/IL-2AK cells in that patient, and that the CDR3 of the TCR V*β*20 was limited. This may in turn suggest the existence of antigen(s), which is(are) processed in antigen-presenting cells during the OK-432 plus IL-2 activation, and which is(are) recognised by the TCRV*β*20 of the effusion lymphocytes. This may also suggest the existence of cross-antigenicity between colorectal adenocarcinoma cells and OK-432. Ogawa *et al* (1993) reported the presence of a common antigen between human gastric cancer cells and OK-432. Bruno *et al* (1996) demonstrated the efficiency of the immune response and showed good clinical outcomes in bladder cancer patients treated with Bacillus Calmette-Guerin (BCG). These researchers also indicated by the analysis of TCR restriction patterns that bladder lymphocytes from patients undergoing BCG treatment were oligoclonal, suggesting a high homology between some BCG antigens and human heat-shock proteins, which are overexpressed in human bladder cancer cells (Bruno *et al*, 1996). This seems very similar to the OK-432 situation shown here. The antigen(s) recognised by the OK/IL-2AK cells is(are) now being addressed.

If lymphocytes expressing the TCRV*β*20, which may recognise the limited antigen(s), are involved in the cytotoxicity of the OK/IL-2AK cells, it is to be investigated whether or not T cells that express a TCR identical to the TCRV*β*20-CDR3 exist in effusion of the other colorectal cancer patients. Then, the CDR3 boundaries of the TCRV*β*20 gene were sequenced and used for the clonotypic PCR analysis of locoregional cells prior to and after OK-432 administration. It was demonstrated that the positive signals of the clonotypic PCR appeared not only in the patient in whom the CDR3 sequences were originally cloned but also in the other colorectal cancer patients, indicating that T cells that express a TCR identical to the cloned TCRV*β*20-CDR3 really exist in effusion of the other patients treated with our immunotherapy. Coulie *et al* (2001) have emphasised the significance of the clonotypic PCR in detecting the response of CTL precursors in the treatment of melanoma patients, mainly because the CTL response is not always as massive as is detectable with tetramer analysis, even in patients with tumours that are regressing after vaccination. We are now investigating the relevance between the clinical responses of OK-432 immunotherapy for effusion and the TCRV*β*20-CDR3 expression in effusion lymphocytes. Only the accumulation of a variety of clinical experiences can prove the significance of the clonotypic PCR using the TCRV*β*20-CDR3 in the OK-432-based immunotherapy.

In summary, locoregional administration of OK-432 alone and OK-432 plus IL-2 was highly effective for the management of malignant effusion from colorectal cancer. OK-432 plus IL-2 induced autologous tumour-reactive CD4+ Th1 killer lymphocytes, which recognised tumour antigen(s) presented with HLA class II molecules on effusion tumour cells by means of preferential usage of TCRV*β*20, and thus possibly contributing to the clinical efficacy of this treatment. Clonotypic PCR analysis using TCRV*β*20-CDR3 sequences may be informative for OK-432-based immunotherapy in treating malignant effusion from colorectal cancer. This possibility is now under investigation, as is the search for OK-432-related tumour antigen(s).
